# Addendum: Dendritic spine geometry and spine apparatus organization govern the spatiotemporal dynamics of calcium

**DOI:** 10.1085/jgp.20181226107312019a

**Published:** 2019-07-31

**Authors:** Miriam Bell, Tom Bartol, Terrence Sejnowski, Padmini Rangamani

Vol. 151, No. 8, August 5, 2019. https://doi.org/10.1085/jgp.201812261.

We would like to clarify that Fig. 9 C was plotted using MATLAB’s built-in “surfc” function (https://www.mathworks.com/help/matlab/ref/surfc.html) and only serves to conceptually represent possible peaks and valleys of calcium AUCs and is not indicative of any experimental or modeling measurements.

**Figure 9.**
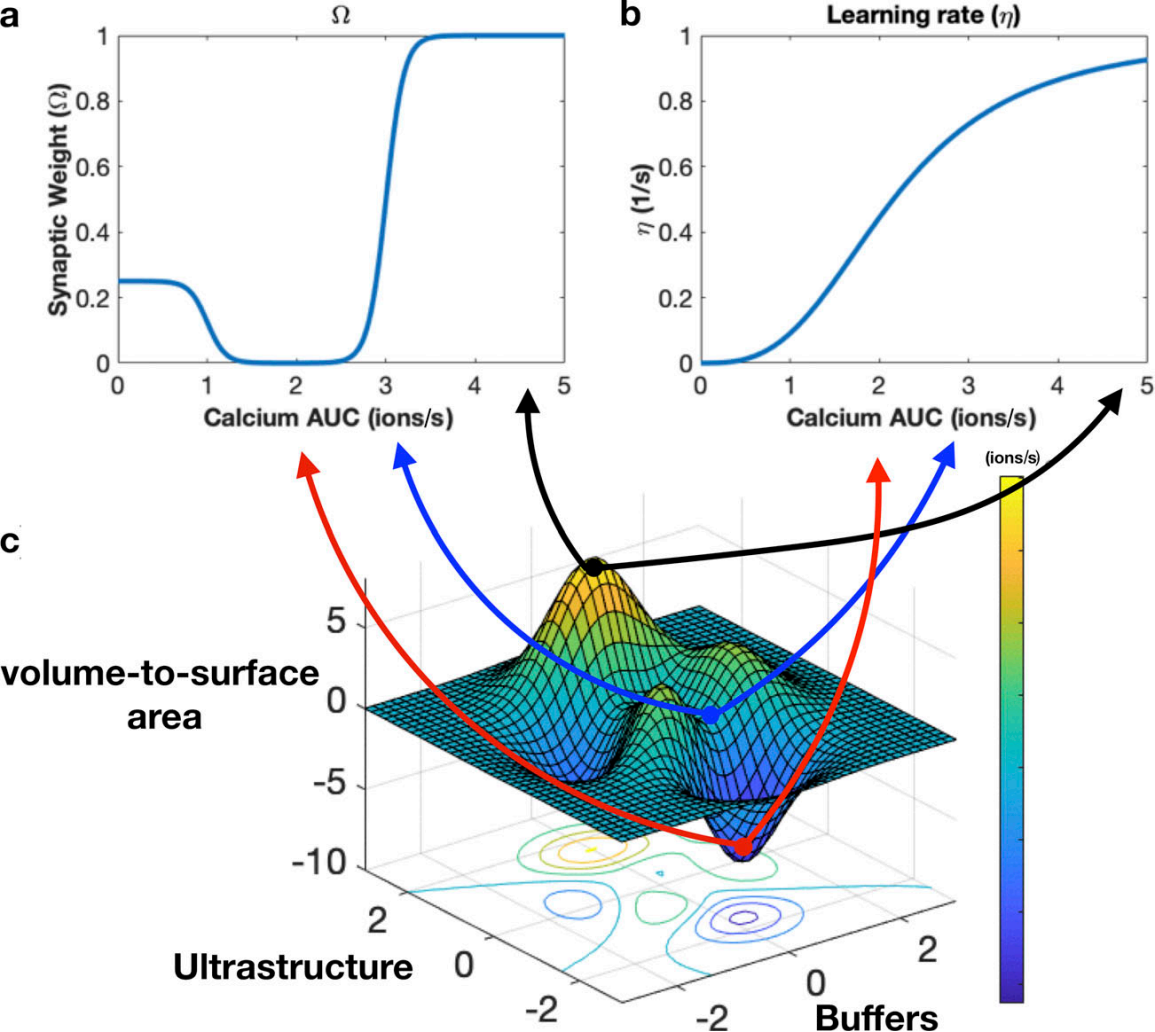
**Biophysical factors can impact synaptic weights through calcium dynamics. (a)** Synaptic weight can be calculated from calcium dynamics (Shouval et al., 2002). We plot changes in synaptic weight due to calcium quantified through accumulated calcium. **(b)** Calcium dynamics also dictate the learning rate of the spine. **(c)** Conceptual representation of the effects of calcium AUC on synaptic weight and learning rate. Using our model, we can map how various factors governing calcium dynamics influence both synaptic weights and learning rates of dendritic spines. This surface plot visualizes how three different factors (volume to surface area ratio, ultrastructure, and calcium buffers) couple to influence calcium AUC (color bar) that feeds back into synaptic weight changes and learning rates.

